# Checkpoint Immunotherapy in Pediatric Oncology: Will We Say Checkmate Soon?

**DOI:** 10.3390/vaccines11121843

**Published:** 2023-12-12

**Authors:** Alexander Ciurej, Elizabeth Lewis, Avanti Gupte, Eman Al-Antary

**Affiliations:** 1 Pediatric Department, Children’s Hospital of Michigan, Detroit, MI 48201, USA; aciurej@dmc.org (A.C.);; 2 School of Medicine, Wayne State University, Detroit, MI 48201, USA; 3 Pediatric Blood and Marrow Transplantation Program, Division of Hematology/Oncology, Barbara Ann Karmanos Cancer Center, Children’s Hospital of Michigan, Detroit, MI 48201, USA; 4 Department of Pediatrics, Central Michigan University College of Medicine, Mt Clemons, MI 48859, USA

**Keywords:** immune, checkpoint, inhibitor, pediatric, oncology, CTLA-4, PD-1, PD-L1

## Abstract

Immune checkpoint inhibitors (ICIs) are a relatively new class of immunotherapy which bolsters the host immune system by “turning off the brakes” of effector cells (e.g., CTLA-4, PD-1, PD-L1). Although their success in treating adult malignancy is well documented, their utility in pediatric cancer has not yet been shown to be as fruitful. We review ICIs, their use in pediatric malignancies, and active pediatric clinical trials, exemplifying some of adult efforts that could be related to pediatric future trials and complications of ICI therapy. Through our review, we propose the consideration of ICI as standard therapy in lymphoma and various solid tumor types, especially in relapsed or refractory (R/R) disease. However, further studies are needed to demonstrate ICI effectiveness in pediatric leukemia.

## 1. Introduction

The historic approach to the treatment of childhood malignancies, consisting of cytotoxic chemotherapy, surgery, and radiation, has left many survivors with lasting adverse effects given the lack of specificity from these traditional approaches. As survivorship of pediatric cancers increases, so does the need for targeted approaches that are both effective and safe. Over the last decade, immunotherapy has made an outstanding contribution in the field of oncology. It is a widely used tool to target cancer cells more precisely by enhancing the host’s immune system. The increased use of various types of immunotherapies has led to a groundbreaking transformation in the field of adult oncology. However, its utility in pediatric malignancies is still in question.

While the immune system consists of many cell types, the T-cell can be thought of as the orchestrators of the immune response. T-cells are activated via two signals. The first involves antigen presentation via the major histocompatibility complex (MHC) on the surface of antigen-presenting cells (APCs) to the T-cell receptor (TCR) and CD4 or CD8 co-receptors [1,2]. Secondly, B7-1/B7-2 receptors on APCs bind CD28 on T-cells [2,3]. Binding of these receptors with their ligands stimulates T-cell proliferation and activation.

**Figure 1:**
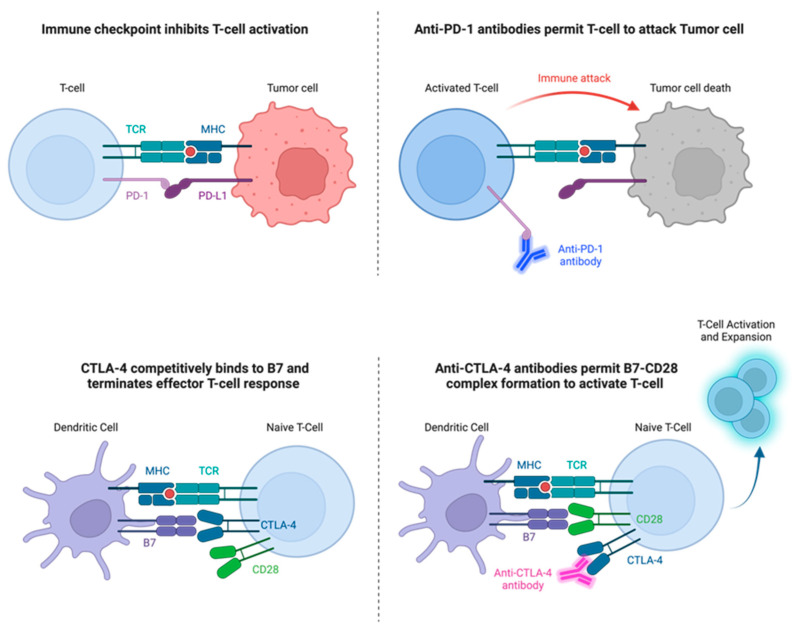
Mechanism of action of immune checkpoint inhibitors. In the **top-left panel**, binding of PD-1 to PD-L1 prevents activation of the primed T-cell, indicated by TCR binding to MHC. In the **top-right panel**, an anti-PD-1 antibody binds to PD-1, allowing the activated T-cell to attack the tumor cell. In the **bottom-left panel**, CTLA-4 binds to B7 on the dendritic cell, which inhibits T-cell activation and clonal expansion. In the **bottom-right panel**, CTLA-4 is bound by an anti-CTLA-4 antibody, allowing the activating CD28 receptor to bind to B7 and subsequent proliferation of effector T-cells. Abbreviations: CTLA-4 = cytotoxic T leukocyte antigen 4; MHC = major histocompatibility complex; PD-l = programmed death protein 1; PD-L1 = programmed death ligand 1; TCR = T-cell receptor.

Conversely, a separate receptor on the T-cell surface, cytotoxic T lymphocyte antigen 4 (CTLA-4), inhibits T-cell activation by interfering with the CD28-B7 complex [4]. CTLA-4 shares 30% homology with CD28 and can bind to B7 with high affinity to outcompete for its binding sites. CTLA-4 terminates the effector T-cell response with the help of regulatory T-cells (Tregs). Tumor cells secrete TGFb among other cytokines that Tregs naturally create to down-regulate an effector T-cell response. High levels of these cytokines in the microenvironment push differentiation of nascent T-cells into Tregs creating a positive feedback loop for tumor survival [4,5]. Tumor cells associated with high Treg concentrations in the microenvironment have poorer clinical outcomes, as they have escaped antitumor immune response and rendered the off-switch ineffective [6,7,8] (Figure 1).

Programmed cell death protein 1 (PD-1) is a receptor found on the T-cell surface which interacts with programmed cell death ligand 1 (PD-L1) on APCs. Like the interaction between CTLA-4 with the CD28-B7 complex, the binding of PD-1 to PD-L1 results in the down-regulation of T-cell activity [4]. Under benign circumstances, PD-1 regulates peripheral tolerance and autoimmunity. However, tumor cells have also been found to utilize PD-L1 in immune evasion. 

Together CTLA-4, PD-1, their respective ligands, and several other receptors are known as immune checkpoints. They can be thought of as the brakes to the immune system. Given their implication in tumor cell immune evasion and cancer progression, they are attractive targets for immunotherapy. Consequently, over the last decade, monoclonal antibodies directed toward CTLA-4, PD-1, and PD-L1 have aimed to counteract the evasion of tumor cells from T-cell cytotoxic activity with clinical success. Moreover, the targeting of other checkpoint inhibitors, such as LAG3 (leukocyte activation gene 3), TIM3 (T-cell immunoglobulin and mucin-domain containing-3), and BTLA (B- and T-lymphocyte attenuator) have shown results in preclinical mouse models, with budding clinical trials showing promise [9].

This review aims to expand and summarize the existing knowledge base of checkpoint immunotherapy use in pediatric oncology.

## 2. Methodology

With keyword searches on PubMed and clinicaltrials.gov, using phrases including “immune checkpoint inhibitor”, “PD-1 inhibitor”, “PD-L1 inhibitor”, “CTLA-4 inhibitor”, “pediatric leukemia”, “pediatric lymphoma”, “pediatric solid tumor”, we found articles to review. No date publication date cutoff was used. Each article was read, and relevant information was extracted and summarized using our own language. In this review, we propose consideration of ICI as standard therapy in lymphoma and various solid tumor types, especially in relapsed or refractory (R/R) disease. However, further studies are needed to demonstrate ICI effectiveness in pediatric leukemia.

## 3. Checkpoint Inhibitor Use in Pediatric Oncology

The first immune checkpoint inhibitor (ICI) to be approved by the Food and Drug Administration (FDA) in 2011 was ipilimumab, a monoclonal antibody that targets CTLA-4 on T-cells [10]. In 2014, pembrolizumab and nivolumab, anti-PD1 monoclonal antibodies, were approved by the FDA [11]. The advent of immunotherapy has led to several advances in the treatment of many adult cancers, notably melanoma, lung, head and neck, kidney, and bladder. However, the pediatric experience has been more limited, largely due to the paucity of mutational burden in pediatric cancers. Given the relative lack of mutations, there are fewer chances for the host immune system to recognize “foreign” tumor antigens. Moreover, many pediatric tumors lack PD-L1 expression, rendering them immunologically cold [12]. Furthermore, the immature immune system of pediatric patients may be insufficient in mounting a robust immune response. While there have been promising results in the treatment of adult malignancies, similar success in pediatric cancer is lagging. Here, we discuss the utility of immune checkpoint therapy in pediatric solid and hematologic malignancies.

### 3.1. Solid Tumors

Pediatric brain and extra-cranial tumors represent nearly half of all pediatric malignancies. Aggressive upfront therapy, often consisting of induction chemotherapy, local control via surgical resection and/or radiation, and consolidation chemotherapy, with or without maintenance therapy, has led to improvements in long-term survival. However, the adverse effects of these therapies have detrimental effects on developing patients. Despite recent advances, the survival among many pediatric patients with solid tumors remains dismal, especially in those with relapsed or refractory (R/R) disease. A review article by Ring et al. describes several opportunities for checkpoint immunotherapy in the treatment of pediatric intra- and extra-cranial solid tumors, namely CTLA-4, PD-1/PD-L1, OX-2 membrane glycoprotein (CD200), and Indoleamine 2,3-dioxygenase (IDO) [13] (Table 1).

#### 3.1.1. Brain Tumors

Central nervous system (CNS) tumors are the most common solid malignancy and the most common cause of cancer-related death in the pediatric population. The use of immune checkpoint therapy among CNS tumors has been limited due to the immune senescence of many brain tumors, the blood–brain barrier impeding penetration, and the paucity of PD-L1 expression [41]. Diffuse intrinsic pontine glioma (DIPG) is one of the most aggressive brain tumors with dismal prognosis. Attempts have been made to use immune checkpoint inhibitors like PD-1 blockade but have been unsuccessful, thought to be due to lack of PD-L1 expression in DIPG [42]. IDO, another immunotherapeutic agent, is implicated in tryptophan metabolism and creates an acquired antigen-specific tolerance in T-cells [43]. Higher expression of IDO correlates with worse prognosis and more metastatic potential of tumors [44]. A phase Ib trial added indoximod, which blocks IDO, to conventional radiation and chemotherapy in patients with DIPG which resulted in improved outcomes and was a well-tolerated regimen [14].

It has been postulated that the two subsets of populations that PD-1 blockade is seen to be effective in are patients with DNA mismatch repair and those with SMARCB1 deletion [45]. Bouffet et al. [46] treated two siblings with DNA mismatch repair who had hyper-mutated, recurrent glioblastoma multiforme (GBM) with the anti-PD-1 inhibitor nivolumab. They reported both clinical and radiological responses in both these patients [46]. Based on these findings, many other institutions have treated patients with DNA mismatch repair and hyper-mutated tumors with nivolumab when other therapies have failed. The first prospective clinical trial (NCT02992964) tested the use of nivolumab in patients with bi-allelic mismatch repair deficiency (bMMRD) and hyper-mutated malignancies (tumor mutation burden more than five mutations/megabase) and included malignant gliomas which resulted in prolonged survival among these patients [15]. SMARCB1-deficient tumors like atypical teratoid rhabdoid tumor (ATRT) are considered mutationally quiet; however, there have been a few isolated reports of using atezolizumab/pembrolizumab/nivolumab in this disease population with a minimal response [47]. These small studies have paved the way for the current trial NCT04416568 (described below in active trials section). 

A single institution retrospective study evaluated a handful of patients with R/R CNS tumors treated with monotherapy or combination of ICIs, including ipilimumab, nivolumab, and/or pembrolizumab. ICI therapy was found to be well tolerated with fair response rates [16]. Another single institution study studied nivolumab in patients with R/R pediatric brain tumors and concluded that immune checkpoint inhibitors have better outcomes in brain tumors with high PD-L1 expression and a high tumor mutation burden as evidenced by a better median survival in those patients who were PD-L1 positive [17].

##### Active Trials in Brain Tumors

A phase II trial conducted by the Pediatric Brain Tumor Consortium is investigating pembrolizumab in patients between 1 and 18 years old with R/R high-grade gliomas, DIPG, hyper-mutated brain tumors, ependymomas, and medulloblastomas who have recovered from prior therapies (NCT02359565) (Table 2). 

NCT04416568 is a phase II trial which assesses the combination of nivolumab/ipilimumab in pediatric patients and young adults with SMARCB1-deficient tumors, including ATRT. 

#### 3.1.2. Neuroblastoma

Neuroblastoma (NB) is the most common extra-cranial solid tumor in children and represents nearly 15% of all pediatric cancer-related deaths. High-risk NB (HRNB) has a very high relapse rate despite the addition of anti-GD2 (ganglioside) immunotherapy; hence, newer modalities in treatment are warranted. A few studies have indicated that PD-L1 is overexpressed in tumor samples of patients with HRNB as well as in lymphocytes obtained from metastatic bone marrow samples. These patients with overexpression of PD-L1 were thought to have a poor prognosis [48,49]. One study suggests that cytokines like interferon gamma, which are up-regulated after treatment with immunotherapy such as the anti-GD2 dinutuximab, in fact induce PD-L1 expression in NB cell lines. This could potentially pave the way for future treatments combining dinutuximab with immune checkpoint inhibitors for HRNB [50]. A case report by Ehlert et al. also showed a complete response and partial response in two HRNB patients treated with a combination of nivolumab and dinutuximab after failing front-line therapy [18]. 

As discussed earlier, most pediatric cancers are immunogenically cold with a very low tumor mutation burden and hence the utility of ICIs may be limited. However, Valind and Gisselsoon postulated that there may be groups of patients within HRNB that do show a higher degree of neo-antigen expression, and these patients could be particularly responsive to ICIs. Their analysis showed that non-MYCN amplified NB patients have a high neo-antigen burden. This group was, however, found to have less PD-1 and PD-L1 expression compared to adult tumors but a degree of CD8+ tumor infiltrating lymphocytes (TIL) that was comparable to adult malignancies [51]. Preclinical NB murine models have demonstrated modest response to PD-1/PD-L1 and CTLA-4 blockade [52,53]. 

NCT01445379 was a phase I clinical trial that evaluated ipilimumab (anti-CTLA-4) in which one NB patient was treated after failing standard therapy. Following treatment with ipilimumab, he had stabilization of disease for two months, after which he was started on other NB directed therapies [19]. Since adult malignancies showed a good response to a combination of PD-L1 and CTLA-4 inhibition, a recently completed pediatric phase I/II trial (NCT02304458) combined ipilimumab and nivolumab to treat R/R sarcomas and included R/R NB. However, only two partial responses were achieved among the 55 patients that were evaluated [45]. 

##### Active Trials in Neuroblastoma

NCT05302921 is an active phase II trial combining cryoablation therapy along with dual checkpoint inhibition with nivolumab and ipilimumab in which patients with NB are included (Table 2).

NCT04412408 is an early phase I study that enrolled patients with HRNB who have failed two lines of therapy and were treated with nivolumab. Results for the study have not yet been published. 

#### 3.1.3. Wilms Tumor

Wilms Tumor (WT) is the most common pediatric renal malignancy with an incidence of nearly 6 per 1,000,000 children in the US [54,55]. WT survival rates exceed 90% in most cases. However, 5–10% of patients will have unfavorable histology defined by anaplasia. Patients with anaplastic WT continue to have poorer event-free survival (EFS) and overall survival. Current treatment strategies, per the Children’s Oncology Group (COG), include upfront nephrectomy whenever possible, radiation, and chemotherapy, with the intensification of chemotherapy for anaplasia. Despite this multimodal approach, 50% of children with anaplastic WT will relapse or progress, reflective of the need for improved treatment strategies [56]. 

Compared to other pediatric solid tumors, the expression of PD-L1 is lowest in WT [57]. Routh et al. conducted a pilot study in which they demonstrated higher PD-L1 in anaplastic WT compared to favorable histology WT. This expression is indicative of the risk of recurrence, especially in favorable histology. Due to a small number of anaplastic WT patients in this study, prognostic significance of PD-L1 in the anaplastic group could not be concluded [58]. This group also performed a nested case-control study and established that favorable histology patients with more than 60% expression for PD-L1 were at a high risk of treatment failure of initial therapy [59]. In a landscape paper by Valind et al., a higher neo-antigen burden was detected in *TP53*-mutated WT, and this subgroup of WT patients may potentially benefit from ICI therapy [51]. 

##### Active Trials in Wilms Tumors

NCT05302921 is an active phase II trial combining cryoablation therapy along with dual checkpoint inhibition with nivolumab and ipilimumab which includes patients with WT (Table 2).

#### 3.1.4. Melanoma

Initially thought to be a rare childhood cancer, melanoma is now increasing in incidence among adolescents [60]. The American Cancer Society estimates localized melanoma to have a greater than 99% five-year survival rate. However, this number falls to 32% for those with metastatic disease, with one study showing as low as a 5% overall survival rate [61], reflective of a glaring need for effective therapies for advanced disease, particularly in pediatric patients. 

In adults, ipilimumab has been FDA approved for metastatic melanoma based on a phase III trial comparing it to a tumor vaccine [62]. However, it has been established that pembrolizumab and nivolumab have shown superior results, as measured by progression-free survival and overall survival, compared to ipilimumab in the treatment of adult melanoma [63,64]. 

Ipilimumab was the first checkpoint inhibitor to be studied in pediatric solid tumors, melanoma included. Due, in part, to the low incidence of pediatric melanoma and resulting low sample size in related clinical trials, the FDA used extrapolation with adult patient data to approve ipilimumab for unresectable or metastatic melanoma in pediatric patients older than 12 years in 2017 [45]. Twelve patients with stage III or IV melanoma non-amenable to surgery were administered ipilimumab monotherapy in a phase I trial. The authors reported no objective improvement in the survival rate for these patients, attributed to the small sample size with a comparably lower incidence of pediatric versus adult melanoma [19], but hoped that their study would serve as a foundation for future trials.

In 2017, Geoerger et al. recruited twelve patients aged 12–18 with untreated, advanced melanoma in a phase II study. Patients were treated with ipilimumab monotherapy at 3 or 10 mg/kg. Three of these patients had at least stabilization of disease at one year. Grade 3 or 4 immune-related adverse events (irAEs) were reported in 63% of those receiving the higher dose with no treatment-related deaths. The study was terminated prematurely due to low enrollment; the authors noted the low incidence of pediatric melanoma as a contributing factor and suggested the inclusion of adolescents in future adult trials [21].

One case study of successful ICI use in pediatric melanoma reported an eight-year-old boy with malignant melanoma secondary to Li-Fraumeni syndrome who was treated with three cycles of ipilimumab monotherapy. Stable disease was reported for three years after initiation before disease progression was discovered, after which he began treatment with pembrolizumab [22].

##### Active Trials in Melanoma

NCT01738139 is an active phase I clinical study to determine optimal dosing and side effect profile of ipilimumab and imatinib in metastatic solid tumors, including melanoma. Patients are required to be at least 15 years of age, and results are expected by early 2024 (Table 2). 

NCT02332668 is an active phase I/II trial exploring the efficacy and safety of pembrolizumab in advanced pediatric melanoma. Initially, patients ages from 6 months to 18 years were included; however, inclusion criteria were adjusted to only include those aged 12–18 years. Results can be expected in 2025.

#### 3.1.5. Sarcomas and Other Solid Tumors

Sarcomas encompass a heterogenous group of solid tumors comprising of bone tumors (osteosarcoma [OS] and Ewing sarcoma [EWS]) and soft tissue tumors, namely rhabdomyosarcoma (RMS) and non-rhabdomyosarcoma. Sarcomas make up approximately 10–15% of childhood cancers [65,66]. Prognosis is based on many factors, including histological subtype, level of metastasis, age at diagnosis, and response to front-line therapy. It is imperative to find newer modalities of treatment for the subset of patients who fail front-line therapy and have R/R disease as conventional second-line chemotherapy comes with toxicities and limited efficacy. 

The immunologic composition of the tumor microenvironment of sarcomas has been found to relate to metastatic potential and prognosis [47,67]. The use of ICI in pediatric sarcomas is limited due to the limited expression of PD-1/PD-L1 in pediatric solid tumors namely OS, RMS, and EWS [68]. Koirala et al. reported that PD-L1 expression is associated with worse five-year EFS in OS [69]. Tumor samples from metastatic sites have been found to express PD-L1 whereas primary tumors did not, potentially elucidating a marker for metastatic potential [70]. Kim et al. studied the distribution of PD-L1 expression in various soft tissue sarcomas and found that it was highest in epithelioid sarcoma, not expressed in mesenchymal chondrosarcoma, and was differentially expressed in about 30–40% cases of RMS and EWS [71]. 

SARC028 was a phase II multicenter trial that evaluated the efficacy of the PD-1 inhibitor, pembrolizumab, in advanced soft tissue and osseous sarcomas. All patients had metastatic disease, and eighty were evaluable for efficacy. Sustained objective responses were seen in 18% of soft tissue sarcomas and only 5% of osseous sarcomas. Of note, response to pembrolizumab was observed in only one patient with OS and zero patients with EWS. Most responses were observed in patients with undifferentiated pleomorphic sarcoma and liposarcoma, sarcomas of adulthood, which could indicate limitations in the pediatric population [23].

There has been research suggesting the significance of CTLA-4 expression among pediatric solid tumors. Specifically, increased CTLA-4+ T-cells in peripheral blood among OS and EWS patients have been reported [72]. Furthermore, surface and cytoplasmic expression of tumor cell CTLA-4 has been reported in NB, RMS, and OS. The first pediatric phase I trial of the CTLA-4 antibody, ipilimumab, included patients less than 21 years with R/R refractory solid tumors (17 patients with sarcomas). The most common adverse events reported were immune-related, and no fatal events were reported. While none of the tumors demonstrated measurable response, notably, those who developed adverse events had improved survival [19]. 

COG conducted a phase I/II trial with nivolumab +/− ipilimumab in patients with R/R solid tumors [NCT02304458]. This study concluded that among 41 patients who received the recommended phase II dose of nivolumab along with ipilimumab, 2 patients (one with RMS and one with EWS) had sustained partial responses and 4 had stable disease [20]. 

A phase II trial, OSTPDL1, studied avelumab, a monoclonal antibody against PD-L1, in adolescents with recurrent/progressive OS. However, this drug did not demonstrate any activity in such patients [24].

##### Active Trials in Sarcomas

NCT02332668 is an active trial that will evaluate the recommended phase II dose of pembrolizumab and the efficacy of this dose in patients less than 18 years old with advanced sarcomas and other cancers (Table 2). 

NCT02813135 is an ongoing European basket trial which includes the use of ICI and will stratify patients with pediatric solid tumors according to their molecular profiling [48]. 

NCT04551430 is an active trial using the combination of ipilimumab and nivolumab along with carbozantinib in patients with metastatic soft tissue sarcomas.

### 3.2. Liquid Tumors

Leukemia and lymphoma are the most common malignancies in pediatric population. Their conventional treatment consists of chemotherapy with and without radiation. In order to minimize long-term effects of conventional therapy and increase survival rates, especially in R/R cases, evaluating and improving ICI therapy is warranted (Table 1).

#### 3.2.1. Leukemia

Acute lymphoblastic leukemia (ALL) is the most common childhood cancer, comprising around 75% of all pediatric cancer diagnoses [73]. Estimates show dramatic improvement in the five-year survival rate for ALL patients to greater than 90% over the last decade compared to nearly 10% in the 1960s [74]. Meanwhile, acute myeloid leukemia (AML) has a comparatively poorer prognosis, with five-year survival rates estimated at 68% in young children and 57% in those aged 15–19 years [75]. Therefore, more efforts are warranted to achieve further success in leukemia cure rates. 

It has been posited that, in addition to mechanisms used by solid tumors (i.e., MHC-1 down-regulation, PD-L1 up-regulation), leukemia cells lend their immune avoidance to limited neo-antigen presentation. The result is a lack of opportunity for effector T-cells to bind, recognize, and respond to cancer cells. Moreover, metabolic by-products of the tumor cell, known as danger-associated molecular patterns (DAMPs), are similarly scarce. Dendritic cells, which function to phagocytose these materials, are unable to fully mature and, consequently, can lead to T-cell tolerance of the tumor cells [76]. Checkpoint inhibitors could play a role in further improving survival rates. However, no immune checkpoint inhibitors have been FDA-approved for treatment in pediatric nor adult leukemia. Therefore, we explore all active efforts in this area and the potential leads for improvement in outcomes.

In 2023, Gao et al. noted the disappointing results of anti-PD-1 monotherapy in R/R AML. Fortunately, they did find that combined therapy with tislelizumab (anti-PD-1 antibody), azacitidine (a hypomethylating agent), and decitabine (a nucleic acid synthesis inhibitor) significantly improved outcomes. Two-thirds of participants exhibited at least remission [25] (Table 1). One phase II trial found pembrolizumab treatment after highdose cytarabine to be an efficacious regimen in adult R/R AML patients, with an overall response rate of 46% among 37 participants [26]. Allogeneic hematopoietic stem cell transplant (HSCT) has been an integral part of treatment of AML since 1957 [77]. Tschernia et al. studied the efficacy of pre-transplant cytarabine and pembrolizumab in 9 AML patients compared to 18 control patients who received allogeneic HSCT alone. Results showed 100% survival at 100 days post-transplant in the experimental group compared to 83% in the control group. Additionally, there was no significant difference in severe graft versus host disease rates between groups, indicating both the safety and efficacy of this regimen [27]. Another phase II study examined the effect of anti-PD-1 therapy after autologous HSCT in post-remission, non-favorable risk AML patients as an alternative to allogeneic HSCT, given the psychosocial challenges that come with the latter (e.g., inability to find a matched donor). Twenty adult patients were enrolled. After a median 80-month follow-up time, 50% of patients remained in complete remission with a 70% survival rate, showing this regimen to be a safe alternative to allogeneic HSCT [28]. These studies lend hope to their efficacy in the pediatric population with further study.

On the contrary, Prebet et al. found a harmful effect of the anti-PD-L1 antibody, atezolizumab, with guadecitabine in the treatment of adult AML. Fourteen of the sixteen patients enrolled died after disease progression during the study, with only one achieving response [29]. Similarly, a lack of clinical benefit was found in a study treating R/R AML adults with azacitidine and the anti-PD-L1 antibody, avelumab. Out of 19 patients, only 2 achieved complete remission [30].

NCT03204188 was a phase II trial for high-risk chronic lymphocytic leukemia, as well as small lymphocytic leukemia in patients 18 years and older. Patients in this single-arm study were treated with conventional therapy (ibrutinib plus fludarabine) with the addition of pembrolizumab. Fifteen patients were included in this study, with all members completing the initial 12-week phase. Five were not able to complete the one-year immunotherapy phase, with four experiencing serious adverse events and one experiencing death. The remaining ten participants entered the follow-up extension study; two did not complete this phase due to adverse events, while four had progressions of disease. These results were posted in an April 2023 update. Similarly, NCT02420912 was a phase II trial investigating ibrutinib and nivolumab combination therapy in a cohort of CLL patients and a cohort of diffuse large B-cell patients who had progression of their CLL (a phenomenon referred to as Richter transformation, or RT). Overall, 3 of 10 in the CLL cohort showed complete remission, while 10 of 24 enrolled in the DLBCL cohort showed clinical response [31]. In their 2017 study, Ding et al. showed that pembrolizumab alone led to a response in 4 of 9 enrolled patients with RT, while 0 of the 16 enrolled with CLL had an objective response [32].

##### Active Trials in Leukemia

One upcoming multicenter phase II clinical trial (NCT04546399) will observe the effect of nivolumab with blinatumomab (a CD3/CD19 bi-specific antibody) versus blinatumomab alone in patients with relapsed B-cell ALL. Those included in the study will be aged 1–31 years who have suffered first-time relapse. As of the last update given in April 2023, the study is on partial FDA clinical hold (Table 2). Another proposed trial (NCT03825367) will determine the efficacy of 5-azacytidine priming plus nivolumab in the treatment of R/R AML. This study is not yet recruiting patients. A phase I trial (NCT02879695) is exploring the efficacy of blinatumomab and nivolumab therapy with or without ipilimumab in pre-B-cell ALL. Patients enrolled must be at least 16 years old. The study is currently active with results pending.

The results of these active and future clinical trials might shed light on the clinical use of checkpoint inhibitors in leukemia in both pediatric and adult populations. 

#### 3.2.2. Lymphoma

Lymphoma is the most common malignancy in those aged 15–19 years [78]. Although there has been much success in the upfront treatment of lymphoma, the use of checkpoint inhibitors has proven pivotal for those with R/R disease. The success of checkpoint inhibitors in lymphoma treatment is explained by the high expressivity of PD-L1 and PD-L2 on the malignant cell surface [79]. Moreover, surrounding macrophages in the milieu also express relatively high levels of PD-L1 [80]. Pembrolizumab has been approved by the FDA for use in pediatric lymphoma, while nivolumab is still only approved as second-line therapy in adults with lymphoma [81,82]. 

Nivolumab is an IgG anti-PD1 antibody and is approved by the FDA as first-line treatment of inherited metastatic colon cancer in patients >12 years of age in combination with ipilimumab [83]. It has also been approved as second-line treatment for many other tumors, including small cell lung cancer, melanoma, and lymphoma. The efficacy and safety dosing of the drug had not been studied in children until the 2020 study by Davis et al. The group conducted the ADVL1412 study on refractory or recurrent malignancies in pediatric patients and adults up to age 30 with solid tumors or lymphomas. In the ten patients with Hodgkin lymphoma (HL) treated with nivolumab, five had stable disease, two had a partial response, and one had a complete response within a mean of 4.5 cycles. Additionally, out of ten patients with non-Hodgkin lymphoma (nHL) treated, one showed a complete response. Tumor samples from enrolled patients were tested with immunohistochemistry to determine relative amounts of PD-L1 expression on the cell surface. Interestingly, of the nine HL samples tested, 100% had significant PD-L1 expression (defined as ≥1% of cells in a sample). Of the eight nHL samples tested, 88% were positive for PD-L1 expression. Conversely, only 7 of 47 non-lymphoma tumors had positive PD-L1 expressivity. The authors suggest that high levels of PD-L1 expressivity on lymphoma tumor cells account for the significant response in those treated. General toxicities were reported, with the most common effects being hematologic in origin, including anemia, neutropenia, lymphopenia, and thrombocytopenia; the most common non-hematologic side effect was fatigue, followed by elevated transaminases [33]. This study suggests that nivolumab is a safe and effective alternative therapy in pediatric lymphoma. However, studies with larger numbers and longer follow-up are needed to evaluate the durability of the response.

In light of the increasing response to nivolumab in pediatric lymphoma, it has been studied in combination with other therapeutic modalities. In 2022, Mei et al. studied the effect of nivolumab induction and nivolumab with ifosfamide, carboplatin, and etoposide (ICE) intensification in R/R HL patients. Participants were administered nivolumab every two weeks for up to six cycles as salvage therapy. If the patient showed complete response, they would proceed to autologous HSCT. If they did not show complete response, they would proceed to ICE therapy before transplant. Of the 43 patients, only 9 required ICE. By study’s end, the overall response rate and complete response rate was 93% and 91%, respectively. The authors reported no unexpected adverse events [34]. Harker-Murray et al. studied the success of nivolumab and brentuximab vedotin (BV) induction in R/R HL patients (median age of 16). They received four cycles of induction, and those without a complete response went on to receive BV and bendamustine intensification. Patients that had a complete response after this would continue with consolidation and autologous HSCT. Out of 44 patients, 59% achieved a complete response after induction, while 94% achieved a complete response by the end of intensification. Of those who only required induction, 18% had serious adverse events (grade 3/4), while those requiring intensification (11 patients) experienced a 27% serious adverse event rate. The one-year progression-free survival rate was 91% [35]. A similar study with a 10-patient pediatric R/R HL cohort showed 100% complete remission rate with this same induction and/or intensification regimen [36]. So far, efforts are suggestive of better outcome when nivolumab is used as bridging or part of combined therapy. Larger studies are required to assess its use in pediatric population.

Pembrolizumab is a selective IgG monoclonal antibody and inhibits PD-L1 on tumor cells [84]. KEYNOTE-087 was a clinical trial which treated 210 patients with R/R HL with pembrolizumab. Three cohorts were included: (1) autologous HSCT patients with subsequent BV treatment; (2) salvage chemotherapy and BV; and (3) autologous HSCT alone. A dose of 200 mg of pembrolizumab every 3 weeks was administered to these patients. In total, 145 (69%) of participants showed a disease response: 47 participants went into complete remission, 98 in partial remission, and 31 with stable disease. The most common treatment-related adverse events were hypothyroidism and fever at 12% and 10%, respectively. Severe adverse effects grade 3 or 4 included neutropenia (2.4%), diarrhea (1%), and dyspnea (1%) [37]. Another study observed successful response to pembrolizumab and vorinostat in patients with R/R HL. Of the 32 patients, 78% had an objective response. Fourteen of these 32 patients were previously refractory to anti-PD-1, indicating the combination to be an option in those difficult to treat patients [38]. These efforts led the FDA to grant accelerated approval of pembrolizumab for the treatment of R/R HL in adult and pediatric patients in March 2017 [85]. Subsequent pediatric studies have been conducted. The KEYNOTE-051 study observed the effect of pembrolizumab on multiple pediatric cancer types. Fifteen patients with R/R HL were included in the international study, with 60% achieving objective response with pembrolizumab monotherapy [39]. Another study which included pediatric patients showed improved survival outcomes in those receiving pembrolizumab prior to HSCT [86]. 

KEYNOTE-204 showed that pembrolizumab effectively and safely improved progression-free survival rates compared with BV, supporting the use of pembrolizumab as the preferred treatment option for patients with R/R HL who have relapsed post-autologous HSCT or are ineligible for autologous HSCT [40]. As a result, in October 2020, the FDA extended the approval of pembrolizumab for the following indications: adult patients with R/R HL and pediatric patients with refractory HL or HL that had relapsed after two or more lines of therapy.

##### Active Trials in Lymphoma

NCT03703050 is now recruiting patients with R/R ALK+ anaplastic large cell lymphoma to determine efficacy of nivolumab as treatment in patients older than 6 months of age. Results are expected in 2028 (Table 2).

A phase I/II trial called RELATIVITY-069 is actively recruiting pediatric and young adult patients with R/R lymphoma to investigate the safety and efficacy of relatlimab (LAG3 inhibitor) with nivolumab. This multicenter trial aims to have 68 participants, with results predicted for 2028 (NCT05255601).

One actively recruiting trial (NCT05772624) is searching for HL patients who are 16-years or older. They aim to test the utility of nivolumab with adriamycin, vinblastine, and dacarbazine (i.e., AVD, a first-line treatment for HL). The study is predicted to be completed by 2024.

A large multicenter study (NCT05675410), which aims to have 1875 participants with HL, will compare outcomes between standard therapy versus standard therapy plus brentuximab vedotin and nivolumab. Standard therapy includes chemotherapy (e.g., bleomycin, etoposide, doxorubicin, etc.) and radiation. The study is set to have results in 2031.

NCT02793466 is an active phase I trial assessing safety of durvalumab, a PD-L1 inhibitor, in pediatric patients with lymphoma, solid tumors, or CNS tumors.

## 4. Monitoring and Complications

The most common complications of ICIs are autoimmune events, known as immune-related adverse events (irAEs). The mechanism by which irAEs occur involves the up-regulation of effector T-cell and down-regulation of Treg function, causing a pro-inflammatory state. This leads to auto-reactivity to healthy organ tissue [87]. Checkpoint inhibitors have been shown to be the underlying cause of over two-thirds of irAEs in cancer treatment [88].

One study estimated that irAEs afflict between 86 and 96% of those taking ICIs. Moreover, between 17 and 59% experience severe grade 3 or 4 side effects, as defined in the Common Terminology Criteria for Adverse Events (CTCAE). Namely, about 17% of those receiving anti-PD-1 monotherapy experienced severe side effects, while upward of 59% who were treated with ipilimumab and nivolumab therapy had grade 3 or 4 toxicities [89].

The high prevalence of immune side effects begs for monitoring guidelines. In 2017, the Toxicity Management Working Group, an initiative formed by the Society for Immunotherapy of Cancer (SITC), released recommendations for monitoring adverse immune events. The group recommends obtaining a pre-treatment complete blood count, comprehensive metabolic panel, thyroid studies, morning cortisol and adrenocorticotropic hormone, hemoglobin A1c, electrocardiogram, echocardiogram (if high-risk), troponin, brain natriuretic peptide, creatine kinase, and fasting lipid profile. These act as a baseline and should be repeated prior to starting subsequent cycles. Evaluation of suspected adverse events varies by organ system. For example, colitis is a common finding in cancer patients and can be infectious or autoimmune in nature while taking checkpoint inhibitors. Work-up should include stool studies (culture, ova, and parasites, *C. difficile* antigens), cytomegalovirus PCR, inflammatory markers, CT imaging, and colonoscopy, if deemed necessary. Pneumonitis is another adverse event that, while not common, is associated with higher mortality. Chest CT, pulmonary function testing, and a six-minute walking test are recommended in the work-up. Patients can develop arthropathy, and when rheumatologic origin is suspected, a basic work-up might include anti-nuclear antibody, rheumatoid factor, cyclic citrullinated peptide antibody, erythrocyte sedimentation rate, C-reactive protein, and MRI. Though rare, neurologic effects can occur with treatment, including neuropathy, transverse myelitis, and aseptic meningitis. Lumbar puncture and brain MRI should be included in the work-up [90].

Management of irAEs involves inhibiting the immune response, namely via corticosteroids. High-dose oral corticosteroids (0.5–2 mg/kg) have been recommended as an effective treatment for more severe events [91,92]. Should steroids and withholding the checkpoint inhibitor prove unsuccessful, stronger immunosuppressants are indicated. For example, there has been success in the treatment of irAE with TNF-alpha and calcineurin inhibitors [92,93].

There are benefits and risks to restarting checkpoint inhibitors in those who have experienced an irAE. In one retrospective study, researchers found that 24% of patients had recurrence of the same adverse reaction, while 26% developed a new reaction in a separate organ system when restarting the same drug [87]. Interestingly, administering a different class of checkpoint inhibitor was found to incur a low rate of recurring irAE. In 2017, Menzies et al. reported that out of 67 patients who developed irAE taking ipilimumab, only 2 had recurrence of the same irAE after starting anti-PD-1 inhibition. However, 34% of them did develop a novel irAE with the treatment. Despite this, the authors claim the reactions were low grade, and that drug class switching is a viable, safe option. It is unclear whether the same can be said when switching from PD-1 to CTLA-4 inhibition, as there are reports that up to 90% of those treated with anti-CTLA-4 therapy develop irAE, compared to only 70% in PD-1/PD-L1 patients [94].

## 5. Conclusions

The immune system is a regulated balance between effector cell activation and inhibition to allow a terminating response to foreign cells while also avoiding the inappropriate targeting of healthy host cells. CTLA-4, PD-L1, and PD-1 are transmembrane receptors which act as “brakes” to the immune response and are known as immune checkpoints. Their activation prevents overstimulation and self-attack. Cancer cells have evolved to use the same maneuver to avoid destruction by the host’s immune system. This has offered a new option for cancer treatment over the last decade with specific antibodies directed at these and other associated receptors. While there has been success in the treatment of adult solid malignancy with immune checkpoint inhibition, its use in pediatric cancer has been more evasive, with some theorizing a relative lack of novel tumor cell mutations to blame. Our review of the available literature illustrates the utility of checkpoint inhibition in pediatric malignancies, primarily in relapsed and refractory lymphomas and solid tumors. We conclude that ICIs could be considered as standard therapy in lymphoma and select solid tumors especially R/R disease, given their clinical success. However, there has not been the same success with ICI in pediatric leukemia, and there is no FDA approval for their use for this indication. There are several active clinical trials which we hope will prove as successful treatment options for pediatric cancer. 

## Figures and Tables

**Table 1:** Descriptions and results of cited published clinical trials, retrospective studies, and case reports, categorized by disease type.

Disease	Trial ID	Description	Result
Brain tumor	NCT02502708	Indoximod, photon radiation, and temozolomide in patients with DIPG	Median OS of 14.5 months and 12-month OS of 61.5% compared to historical 10.8 months and 45.3%, respectively [14]
	NCT02992964	Patients with pediatric solid tumors with high mutation burden and mismatch-repair deficiency treated with nivolumab	Two-year OS of 50%; 3 patients with refractory malignant gliomas with CR at time of publication [15]
	PMID: 32627129	Retrospective single institution study of patients with R/R CNS tumors treated with ipilimumab, nivolumab, and/or pembrolizumab	Median duration of treatment of 6.1 months and 7 of 11 patients discontinuing secondary to progression [16]
	PMID: 30681550	Retrospective single institution study of patients with R/R CNS tumors treated with nivolumab	Median time to progression of 5.5 weeks; 3 patients showed PR; median survival for PD-L1+ patients vs. PD-L1 patients was 13.7 weeks vs. 4.2 weeks, respectively [17]
Neuroblastoma	PMID: 32414861	Two patients with refractory HRNB treated with nivolumab and dinutuximab	The first treated for 10 months with CR for 6 months, and the second treated for 9 months with CR in soft tissue lesions and regression of skeletal lesions with treatment ongoing at time of publication [18]
	NCT01445379	Phase I trial of patients with advanced solid tumors treated with ipilimumab with 1 patient with NB	No reported response [19]
	NCT02304458	Phase I/II trial of patients with R/R solid tumors treated with nivolumab plus ipilimumab (ADVL1412) with 1 patient with NB	PD [20]
Wilms Tumor	NCT02304458	Phase I/II trial of patients with R/R solid tumors treated with nivolumab plus ipilimumab (ADVL1412) with 2 patients with Wilms tumors	PD [20]
Melanoma	NCT01445379	Phase I trial of patients with advanced solid tumors treated with ipilimumab with 12 patients with unresectable stage IIIc or IV melanoma	One patient with prolonged SD [19]
	NCT01696045	Phase II trial of patients with stage III or IV malignant melanoma treated with ipilimumab	2 of 8 patients on 10 mg/kg with PR, and 1 of 4 patients on 3 mg/kg with SD; study discontinued due to slow accrual [21]
	PMID: 26647899	Patient with Li-Fraumeni with metastatic malignant melanoma treated with ipilimumab	SD and PFS of 3 years [22]
Sarcoma	NCT02301039	Phase II trial of patients with soft-tissue and bone sarcomas treated with pembrolizumab (SARC028)	7 of 40 patients with soft-tissue sarcoma with objective response (4 undifferentiated pleomorphic sarcoma, 2 liposarcoma, and 1 synovial sarcoma) and 2 of 40 patients with bone sarcoma with objective response (1 OS, 1 chondrosarcoma) [23]
	NCT01445379	Phase I trial of patients with advanced solid tumors treated with ipilimumab	17 patients with sarcomas with no reported response [19]
	NCT02304458	Phase I/II trial of patients with R/R solid tumors treated with nivolumab plus ipilimumab (ADVL1412) with 11 RMS, 14 EWS, 1 myxoid liposarcoma, 13 OS, and 1 synovial sarcoma	1 PR in RMS, 1 PR in EWS, 2 SD in RMS, and no activity in OS [20]
	NCT03006848	Phase II trial of patients with R/R OS treated with avelumab (anti-PD-L1) (OSTPDL1)	Median PFS of 8 weeks and 16-week PFS of 0% [24]
Leukemia	NCT04541277	Tislelizumab with DNA hypomethylation agent +/− CAG in R/R AML	ORR of 63% [25]
	NCT02768792	Pembrolizumab given after high dose cytarabine in R/R AML	ORR of 46% and composite CR rate of 38% [26]
	NCT02768792	Pembrolizumab and cytarabine pre-allogenic stem cell transplant versus transplant alone in AML	No statistical difference in one-year survival rate (67% versus 78%) [27]
	NCT02771197	Pembrolizumab after autologous hematopoietic stem cell transplant in non-favorable risk AML	Two-year LFS of 48% and two-year OS of 68% [28].
	PMID: 35491816	Atezolizumab with guadecitabine in R/R AML	14 of 16 patients died during the trial from disease progression or adverse events, resulting in study termination [29]
	NCT02953561	Azacitidine and avelumab in adults with R/R AML	Overall CR rate was 2/19, calling into question its clinical benefit [30]
	NCT02420912	Nivolumab plus ibrutinib in CLL, DLBCL with RT	3 of 10 CLL patients with CR and 10 of 24 DLBCL patients with clinical response [31]
	NCT02332980	Pembrolizumab in CLL and RT patients	4 of 9 RT patients with clinical response and 0 of 16 CLL patients showed response [32]
Lymphoma	NCT02304458	Nivolumab +/− ipilimumab in pediatric R/R lymphoma	8 of 10 HL patients with objective response and 1 of 10 nHL patients with CR [33]
	NCT03016871	Nivolumab +/− ifosfamide, carboplatin, etoposide intensification in R/R HL	ORR and CR rate of 93% and 91%, respectively [34]
	NCT02927769	Nivolumab and brentuximab +/− bendamustine in R/R HL	Increase of complete metabolic response rate from 59% to 94% after adding bendamustine to intensification regimen [35]
	PMID: 37583696	Nivolumab and brentuximab +/− bendamustine in R/R HL	10 of 10 patients with CR prior to consolidation [36]
	NCT02453594	Pembrolizumab in R/R HL	145 of 210 patients with objective response [37]
	NCT03150329	Pembrolizumab plus vorinostat in R/R DLBCL, follicular lymphoma, or HL	14 of 32 patients with R/R HL previously refractory to anti-PD-1 therapy with objective response [38]
	NCT02332668	Pembrolizumab in R/R HL and other pediatric cancers	9 of 15 with R/R HL with objective response [39]
	NCT02684292	Comparing pembrolizumab versus brentuximab in R/R HL	Median PFS of 13.2 and 8.3 months, respectively, which was statistically significant [40]

AML = acute myeloid leukemia; CLL = chronic lymphocytic leukemia; CR = complete response; DIPG = diffuse intrinsic pontine glioma; DLBCL = diffuse large B-cell lymphoma; EWS = Ewing sarcoma; HL = Hodgkin lymphoma; HRNB = high-risk neuroblastoma; LFS = leukemia-free survival; NB = neuroblastoma; nHL = non-Hodgkin lymphoma; ORR = overall response rate; OS = overall survival or osteosarcoma; PD = progressive disease; PD-L1 = programmed death ligand-1; PFS = progression-free survival; PR = partial response; R/R = relapsed refractory; RMS = rhabdomyosarcoma; RT = Richter transformation; SD = stable disease.

**Table 2:** Immune checkpoint inhibitors (ICI) with their mechanism of action and active clinical trials in specific pediatric oncology tumor types (clinicaltrial.gov; URL accessed 1 July 2023).

ICI Drug and Mechanism of Action	Active NIH Clinical Trial IDs	Study Title
Pembrolizumab (PD-L1 Inhibitor)	NCT02332668	A Study of Pembrolizumab (MK-3475) in Pediatric Participants with an Advanced Solid Tumor or Lymphoma (MK-3475-051/KEYNOTE-051)
	NCT02359565	Pembrolizumab in Treating Younger Patients with Recurrent, Progressive, or Refractory High-Grade Gliomas, Diffuse Intrinsic Pontine Gliomas, Hypermutated Brain Tumors, Ependymoma or Medulloblastoma
	NCT03605589	Pembrolizumab + Blinatumomab Combination in Pediatric and Young Adult Patients with Relapsed/Refractory Acute Leukemia or Lymphoma
Nivolumab (PD-L1 Inhibitor)	NCT02813135	European Proof-of-Concept Therapeutic Stratification Trial of Molecular Anomalies in Relapsed or Refractory Tumors (ESMART)
	NCT03703050	Nivolumab for Pediatric and Adult Relapsing/Refractory ALK+, for Evaluation of Response in Patients with Progressive Disease (Cohort 1) or as Consolidative Immunotherapy in Patients in Complete Remission After Relapse (Cohort 2) (NIVO-ALCL)
	NCT02992964	Pilot Study of Nivolumab in Pediatric Patients with Hypermutant Cancers
	NCT03825367	Nivolumab in Combination with 5-azacytidine in Childhood Relapsed/Refractory AML
	NCT04416568	Study of Nivolumab and Ipilimumab in Children and Young Adults with INI1-Negative Cancers
	NCT04546399	A Study to Compare Blinatumomab Alone to Blinatumomab with Nivolumab in Patients Diagnosed with First Relapse B-Cell Acute Lymphoblastic Leukemia (B-ALL)
	NCT05255601	A Study to Evaluate the Safety, Tolerability, Drug Levels, and Preliminary Efficacy of Relatlimab Plus Nivolumab in Pediatric and Young Adults with Hodgkin and Non-Hodgkin Lymphoma (RELATIVITY-069)
	NCT05302921	Neoadjuvant Dual Checkpoint Inhibition and Cryoablation in Relapsed/Refractory Pediatric Solid Tumors
	NCT05675410	A Study to Compare Standard Therapy to Treat Hodgkin Lymphoma to the Use of Two Drugs, Brentuximab Vedotin and Nivolumab
	NCT05772624	Low-dose Nivolumab in Combination with AVD as Front-Line Therapy for Classic Hodgkin’s Lymphoma
Durvalumab (PD-1 Inhibitor)	NCT02793466	Durvalumab in Pediatric and Adolescent Patients
Ipilimumab (CTLA-4 Inhibitor)	NCT01738139	Ipilimumab and Imatinib Mesylate in Advanced Cancer
	NCT02879695	Blinatumomab and Nivolumab with or Without Ipilimumab in Treating Patients with Poor-Risk Relapsed or Refractory CD19+ Precursor B-Lymphoblastic Leukemia
	NCT04416568	Study of Nivolumab and Ipilimumab in Children and Young Adults with INI1-Negative Cancers
	NCT05302921	Neoadjuvant Dual Checkpoint Inhibition and Cryoablation in Relapsed/Refractory Pediatric Solid Tumors
